# Ceiling effect in EMR system assimilation: a multiple case study in primary care family practices

**DOI:** 10.1186/s12911-017-0445-1

**Published:** 2017-04-20

**Authors:** Marie-Claude Trudel, Josianne Marsan, Guy Paré, Louis Raymond, Ana Ortiz de Guinea, Éric Maillet, Thomas Micheneau

**Affiliations:** 1 Information Technology Department, HEC Montréal, 3000, Chemin de la Côte-Sainte-Catherine, Montréal, Québec H3T 2A7 Canada; 2 Université Laval, Québec, Canada; 3 Université du Québec à Trois-Rivières, Trois-Rivières, Canada; 4 Université de Sherbrooke, Longueuil, Canada

**Keywords:** Electronic medical record, Assimilation phase, Ceiling effect, Primary care

## Abstract

**Background:**

There has been indisputable growth in adoption of electronic medical record (EMR) systems in the recent years. However, physicians’ progress in using these systems has stagnated when measured with maturity scales. While this so-called ceiling effect has been observed and its consequences described in previous studies, there is a paucity of research on the elements that could explain such an outcome. We first suggest that in the context of EMR systems we are in presence of a “tiered ceiling effect” and then we show why such phenomenon occurs.

**Methods:**

We conducted in-depth case studies in three primary care medical practices in Canada where physicians had been using EMR systems for 3 years or more. A total of 37 semi-structured interviews were conducted with key informants: family physicians (about half of the interviews), nurses, secretaries, and administrative managers. Additional information was obtained through notes taken during observations of users interacting with their EMR systems and consultation of relevant documents at each site. We used abductive reasoning to infer explanations of the observed phenomenon by going back and forth between the case data and conceptual insights.

**Results:**

Our analysis shows that a ceiling effect has taken place in the three clinics. We identified a set of conditions preventing the users from overcoming the ceiling. In adopting an EMR system, all three clinics essentially sought improved operational efficiency. This had an influence on the criteria used to assess the systems available on the market and eventually led to the adoption of a system that met the specified criteria without being optimal. Later, training sessions focussed on basic functionalities that minimally disturbed physicians’ habits while helping their medical practices become more efficient. Satisfied with the outcome of their system use, physicians were likely to ignore more advanced EMR system functionalities. This was because their knowledge about EMR systems came almost exclusively from a single source of information: their EMR system vendors. This knowledge took the form of interpretations of what the innovation was (know-what), with little consideration of the rationales for innovation adoption (know-why) or hands-on strategies for adopting, implementing and assimilating the innovation in the organization (know-how).

**Conclusions:**

This paper provides a holistic view of the technological innovation process in primary care and contends that limited learning, satisficing behaviours and organizational inertia are important factors leading to the ceiling effect frequently experienced in the EMR system assimilation phase.

## Background

It is widely recognized that information systems (IS) have great potential to reduce healthcare costs and improve clinical outcomes [1]. However, the full materialization of this potential is yet to be seen, considering how far behind hospitals and medical practices are in their technological sophistication compared to organizations in other sectors such as manufacturing, financial services and transportation [2, 3]. The healthcare industry’s lag behind other industries in innovating with information technology (IT) is attributed to the unique challenges and barriers faced by this industry [4]. In other words, healthcare is different from other sectors with which it is analogized in regard to its development of IT-enabled capabilities. For instance, the healthcare industry is fragmented and decentralized to a much higher degree than most others, it faces a much stricter regulatory environment, requires complex business models (e.g. unique customer and pricing constraints), and many of its inputs, products and services cannot be standardized [5]. Nonetheless, industry analysts recently stated that healthcare providers around the world have an opportunity to harness high-impact IT innovations to accomplish the necessary transformation of this sector towards a patient-centric delivery model [6].

Over the last few years, primary care physicians have been encouraged to invest in and use electronic medical record (EMR) systems^1^ through various incentive programs (e.g., the HITECH Act in the United States or MEDCOM in Denmark) [7, 8]. An EMR is a type of information system that can support both administrative and clinical tasks. On the administrative side, such a system aims to alleviate the logistical problems associated with managing paper records. On the clinical side, an EMR can improve the quality of clinical decisions with the help of built-in evidence-based advices and decision-support algorithms. Such a system can also facilitate communication among healthcare providers within a single- or between several organizations. It is believed that using such systems will improve the quality and safety of patient care and enhance the performance of primary care physicians, in particular by facilitating communication and exchanges of information about patients [9]. However, contradictory results are found in the extant literature showing that after an EMR is acquired, the quality and safety of care may improve, remain stable or even decline [10, 11]. The same can be said for clinicians’ performance in terms of efficiency gains, which are seemingly slow to materialize [12–14]. While administrative and clerical duties are often fully supported by EMRs in healthcare settings, the same cannot be affirmed of clinical duties [14].

Viewed as IT artifacts, EMRs have been the subject of empirical studies that focus on one or more of the four phases suggested by Swanson & Ramiller [15] to describe the IT innovation process. In the first phase – comprehension – the goal is to give meaning to the EMR and justify its acquisition by a medical practice. In the second phase – adoption – a decision is made to commit resources to the EMR project. In the third phase – implementation – the choices and the actions that shape the deployment of an EMR are determined and applied. The fourth and last phase – assimilation – begins when the EMR is used on a daily basis and continues until complete and transparent integration of the EMR into the organization [15].

This last phase, which is the focus of the present study, was recently investigated by Lanham et al. [16], Paré et al. [17] and Raymond et al. [14], in primary and ambulatory care settings. One important conclusion of this stream of research is that “extended” or “sophisticated” use of an EMR leads to more individual and organizational benefits than “basic” use (e.g., [14]). Sophisticated use refers to interactions with the system in line with the higher maturity “stages” found in frameworks such as the Electronic Medical Record Adoption and Maturity Model from Canada’s Health Informatics Association [18], or the Electronic Medical Record Adoption Model from the American Healthcare Information and Management Systems Society [19], which assume from a national perspective that higher maturity leads to optimized patient care and ultimately, to improved public health. Some of the functionalities associated with such maturity levels are presented in Table 1 [18].

**Table 1:** Overview of EMR functionalities for maturity levels 1, 3 and 5

Functional Category	Description		
	Level 1	Level 3	Level 5
Practice Management	Hybrid scheduling in place. Billing is printed daily. EMR used to communicate within practice.	Advance appointment tracking with EMR. EMR generates billing-related reports. Use EMR to track tasks directly related to patient record.	Appointments are managed online and linked to EMR scheduler. EMR autopopulates billing codes based on encounter notes. Communicate with other providers through EMR.
Information Management	EMR used to input patient demographics, patient visit, and encounter notes. Use varying local codes and non-standard nomenclature. Scan paper-based documents with written instructions and notes into EMR.	EMR identifies sub-population for recurrent visits or proactive care; enter clinical data for focused patients. Agreed upon nomenclature for coding standards and charting. Written protocol.	Use EMR to co-manage and reconcile patient enrollment. Send and or/receive data of individual patient records from one EMR to another. Advanced nomenclature coding standards.
Patients Results Management	Laboratory, digital imaging, hospital information is scanned into the EMR. EMR prepopulate the generic referral/consult templates. Paper-based referral reviewed for specialist.	EMR to do advanced tracking and management of laboratory and imaging results. Review and analyze information from hospital. Advanced tracking and management of referrals and consults.	EMR to generate longitudinal lab, imaging, and information analysis from different care settings. Patients manage online appointment booking.
Diagnosis Support	Follow-up care and resources are combination of EMR and paper.	EMR generates recommendations for patient assessment tools. Autopopulate regional registries and do advanced tracking of preventative care such as proactive profiling. EMR updated with emerging, changing, and appropriate evidence and guidance.	EMR linked to regional health record for most effective diagnostic procedures. EMR linked to regional repository to access provider-specific preventative/follow up care. EMR aggregated database to conduct real time analysis of de-identifiable data.
Treatment Planning Support	Subjective, Objective, Assessment and Plan (SOAP) notes for care planning and coordination. EMR is used to create basic prescription and renewals. EMR manages at least one chronic condition using templates for chronic disease management (CDM).	Customized templates for care planning and coordination. Customized prescription creation. Manage multiple chronic conditions using customized templates/forms.	EMR to access regional registries for care planning and coordination. EMR to share information with regional disease registries regarding CDM. EMR linked to regional system for medication management.
Patient Engagement and Communication	Educate patients via EMR screen and input patient results into EMR with scanned copies.	EMR develops customized educational modules. Set up customized templates for self-care/co-management.	Patients have access to regional web portals for education and self-management.
Evaluation and Monitoring	EMR to set up system-wide alerts and reminders for health outcomes. EMR to generate reports on infectious diseases.	EMR to monitor Health Quality Indicators (HQI), Health Outcomes (HO), and Public Health, and generate reports.	EMR receives information from regional reporting system regarding health outcomes. Generate up-to-date information based on symptoms for public health. Receive information to define sub-population for health quality indicators.

Empirical studies of EMR adoption maturity are generally based on factor models, in which the scope of the system’s use is compared with its actual or perceived benefits, without accounting for what may or may not have helped attain these benefits. Interestingly, these studies also reveal that the shorter the EMR had been in use in a healthcare organization, i.e. the shorter the assimilation phase, the higher the number of advanced users [17]. Put differently, prior research shows that EMR usage levels out with time at a low maturity level.

Price et al. [20] refer to this stagnation in EMR assimilation, represented by a low maturity level and weak untapped benefits, as a ceiling effect. Our review of the extant literature reveals that no prior study has attempted to identify individual and/or organizational factors explaining the presence of such an effect. In this line of thought, the present study seeks to fill this gap by attempting to answer the following question: *Why do ceiling effects occur in the assimilation phase of EMR systems in primary care medical practices?*

## Conceptual framework

Price et al. [20] use the term “ceiling effect” to refer to stagnation in EMR assimilation. To better understand the concept of ceiling effect and how it has been applied in other domains, we reviewed the extant literature and found that this term has three distinct meanings in the fields of psychology and education. The first definition is “an intervention having limited effect because the population is already at, or near, a pinnacle point”, that is, everybody has substantial mastering of the topic assessed ([21], p. 958). The second definition is “the limitation of an assessment to capture the extent and variance of accomplishment because essentially the assessment is too simplistic” and everybody scores very high ([21], p. 958). Both these definitions consider the ceiling effect as a measurement problem [21, 22] that limits the ability to make improvements since everyone seems an expert [23, 24]. In health informatics research, such measurement challenges may be the reason for mixed results obtained in assessing the effect that electronic health records (EHRs) have on quality improvement in US hospitals [25, 26]. A third definition considers the ceiling effect as “a maximum attainable score given students’ background and available information” [21].

This last definition represents an “intermediary ceiling” or “tiered ceiling” that learners may be confronted with [21, 27]. To illustrate the notion of tiered ceiling effect, Judson [21] gives the example of a test measuring advanced understanding of nanotechnology administered to novices as well as nanotechnology experts. It is foreseen that the novices will be far outscored by the experts because they are limited by their generalist background and surface understanding of fundamental nanotechnology concepts. However, if given an overview of nanotechnology, the novices would all end up at almost the same level of comprehension (i.e., a tiered ceiling), while still scoring below the experts. This type of ceiling effect is not an artifact of assessment limitations, but of “learner parameters” or “the boundaries of the learners within the observed timeframe and environment” ([21], p. 960). Such authentic ceiling effects are most likely to occur for phenomena with which people have minimal familiarity and about which they have minimal and uniform information from a very limited number of sources [22]. In sum, a tiered ceiling effect occurs when the learner is not yet an expert in a given topic, and barriers are constraining him or her to further learn about that subject.

In light of the studies in psychology and education on the notion of ceiling effect, our interpretation of Price et al.’s [20] use of this concept is that in the context of EMR assimilation we are in fact in the presence of a tiered ceiling effect. Therefore, we adapt Judson’s [21] definition to suggest a definition of the EMR tiered ceiling effect as *a maximum attainable level of EMR assimilation given EMR users’ boundaries and available information*. In other words, EMR assimilation stops at some level because of constraining conditions on the users, even if higher levels of assimilation could be reached.

Based on Price et al. [20] and theory about the ceiling effect in the fields of psychology and education [21–24, 27] we make the following proposition: *In primary care medical practices, a tiered ceiling effect in EMR assimilation is created by a series of constraining conditions that occur or build up throughout the four phases of the IT innovation process*. When analyzed end to end, the innovation process briefly described in the previous section can be seen as a series of decisions, each of which influences subsequent decisions, such that certain choices made early in the process may have unexpected consequences on subsequent phases. For example, Yang et al. [3] showed that the way in which awareness was driven in the earliest stages of the innovation process for a vital signs monitoring system led to a chain of discrete events (e.g. decisions on the type of project leader, selection of project team members). These authors indicate, for each stage and sub-stage, which conditions or contextual issues contribute to a beneficial result. With this in mind, we decided to investigate the whole IT innovation process, as it relates to EMRs, in order to gain a better understanding of the conditions leading to ceiling effects and, consequently, to a failure to attain the expected or purported benefits from these systems.

Finding how and why ceiling effects occur in the assimilation of EMRs within primary care medical practices is important and timely because healthcare professionals recognize that this phenomenon is occurring, and that discontinuing the use of EMRs is not an option [11, 20]. There is growth in EMR adoption, with recent adoption rates of 73% in Canada and 84% in the United States [28]. However, physicians’ progress in EMR use is slow after adoption [14, 29]. Overcoming ceiling effects is crucial since EMRs are becoming an important part of a larger electronic healthcare ecosystem and have the potential to provide many administrative benefits and, more importantly, to support clinical decisions and enhance inter-professional collaboration [11].

Our contributions are both theoretical and practical in nature. On a theoretical level, our research offers a clear definition of – and a plausible explanation for – the ceiling effect observed in EMR assimilation. On a practical level, our empirical investigation sheds light on the constraints that can arise and be created during the EMR innovation process and that may hamper the full assimilation of EMRs in primary care medical practices, which could help prevent the development of detrimental ceiling effects.

## Methods

As Price et al. [20] observed considerable ceiling effects in the assimilation of EMRs in Canadian primary care medical practices, we have chosen to study such practices to answer our research question. Note that following our investigation’s data collection phase, quantitative studies have further shown the presence of the ceiling effect detected by Price et al. [20] in Canada [17, 30, 31]. To provide a deeper understanding of elements leading to a ceiling effect in EMR usage, we conducted a qualitative research using a multiple case study method. Case studies are employed to understand the dynamics of a contemporary phenomenon within single settings, especially when it is difficult to delineate between the phenomenon and its context [32]. More specifically, we studied private medical clinics throughout their IT innovation processes to understand how and why ceiling effects occurred in the assimilation of their EMR. Our unit of analysis is the EMR innovation process in primary care medical practices. This study received approvals from the ethics board committees of the participating universities.

A quantitative survey preceded this study and found that a majority of primary care practices did not use most of their advanced EMR functionalities, despite their availability [31]. We were therefore confident to find a ceiling effect in any site we would approach. Three medical practices, which we have named Alpha, Delta and Epsilon to preserve their anonymity, were identified as “experienced” EMR user organizations by EMR providers and Canada Health Infoway. The medical practices all operate within the same Canadian province and are obliged to follow the same governmental regulations on healthcare provision and remuneration. From a theoretical sampling standpoint [33] we wanted to study more experienced clinics in order to appreciate the “height” of the ceiling, or the maximum attainable level of EMR assimilation given EMR users’ boundaries and available information in Canada. As we looked for variance in the brand of EMR solutions that the medical practices adopted, we investigated three clinics whose EMRs are provided by three different software vendors. We also looked for variance in the number of years since adoption. The systems had been in use for 5 to 9 years, depending on the clinic. The three clinics are also of different sizes in terms of number of physicians, nurses and administrative employees, as well as city population. The medical director of each clinic was contacted by phone and agreed to participate in the study. Table 2 presents the profiles of the participating sites.

**Table 2:** Profiles of the primary care medical practices

	Alpha	Delta	Epsilon
Number of years since initial EMR deployment	9	7	5
EMR solution in use	EMR-A	EMR-B	EMR-C
Number of physicians	6–10	11–15	15–20
% of physicians using the EMR solution	99%	100%	100%
Number of nurses	1–5	6–10	1–5
% of nurses using the EMR solution	100%	100%	100%
Number of administrative employees	1–5	16–20	6–10
% of administrative employees using the EMR solution	100%	100%	100%
Location of the clinic	Small city	Medium city	Large city

Each medical practice was visited by a team of three researchers for an average of two full working days. At each clinic, semi-structured interviews were conducted with physicians (about half of the interviews) as well as nurses, secretaries and administrative managers. Table 3 provides an overview of the topics that were part of our interview guide.

**Table 3:** Topics covered during the interviews in medical practices

Topic	Types of interviewees
Socio-demographics of the interviewee	All interviewees
Socio-demographics of the clinic	• Administrative managers • Clinical managers
History of the clinic	• Administrative managers • Clinical managers
Brand of EMR and functionalities available in the chosen EMR	• Administrative managers • Clinical managers
EMR innovation process in the clinic	All interviewees
Changes to the clinic’s functioning brought by the EMR	All interviewees
Changes to the interviewee’s work brought by the EMR	All interviewees
Interviewee’s use of the EMR (evolution through time as well as current use) including the number of the EMR functionalities utilized	All interviewees
Interviewee’s satisfaction with EMR use	All interviewees
Individual and organizational benefits of the EMR	All interviewees

The extent to which and how the different available EMR functionalities were actually used was mainly determined on the basis of self-reported use by interviewees and observations of users interacting with their EMR. As shown in Table 4, a total of 37 semi-structured interviews were conducted with key informants, tape-recorded and transcribed. The average interview length was 45 minutes. Between interviews, additional information was obtained through field notes, taken from observations of clinicians and administrative staff interacting with the EMRs, and the consultation of relevant documents (e.g., user manuals) at each site. This combination of multiple sources of information allowed converging lines of inquiry to develop through data triangulation [34].

**Table 4:** Detailed information on data collection

	Alpha	Delta	Epsilon
Length of site visit	3 days	2 days	2 days
Number of interviews	13 interviews: 1 administrative manager, 4 secretaries, 3 nurses, 5 physicians	12 interviews: 1 administrative manager, 5 secretaries, 1 nurse, 5 physicians	12 interviews: 1 administrative manager, 5 secretaries, 1 nurse, 5 physicians
Total number of hours of interviews	8	10	5
Number of pages of verbatim	320	353	213
Field notes	✓	✓	✓
Documents	✓	✓	✓

In terms of data analysis, abductive reasoning was used to combine both deduction and induction in an iterative fashion [35]. In other words, we inferred an explanation of the observed phenomenon by going back and forth between the case data and the conceptual insights [36]. The portrayal by methodological scholars [36] of "interpretation as a characteristically abductive exercise" required that we remained open to the use of emergent theoretical concepts and models for interpretive discovery purposes.

At the end of each site visit, members of the research team met in order to discuss the newly collected material and conduct a debriefing. A first case was coded independently by three researchers. Coding discrepancies were discussed and resolved during a team meeting. The other cases were subsequently coded by one of the researchers and validated by another team member. NVivo software was used to perform the coding process. The codes used corresponded to the four phases of the innovation process presented earlier [15] and to constraining conditions on the users in each phase [21], including the influential sources of information that can potentially represent barriers to assimilation [22].

As suggested by Miles and Huberman [33], we synthesized the extracted data using both chronological and schematic matrices. This allowed us to depict the EMR innovation process in each setting and develop narrative stories of 20 pages each (not shown here). Ultimately, our three cases were compared and contrasted to uncover trends and patterns. Following Langley’s data analysis approach [37], the application of abstraction and generalization principles allowed us to infer a process explanation of the clinics' ceiling effect in their assimilation of EMR.

## Results

In this section, we first show that an EMR tiered ceiling effect is present in each of the three clinics. Then, we describe the main decisions and activities related to the EMR innovation process in each clinic. Finally, we show that a set of constraining conditions on the EMR users existed in the three clinics and led to the tiered ceiling.

### Evidence of EMR tiered ceiling effect in the primary care family practices

**Fig. 1:**
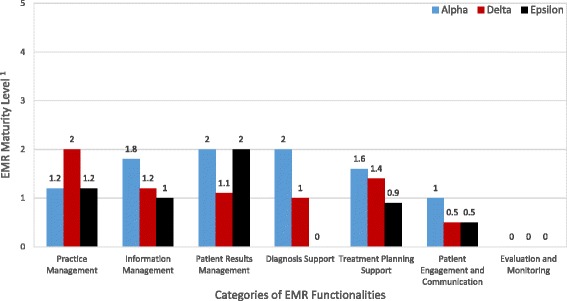
Tiered ceiling effect in EMR assimilation. ^1^EMR maturity models are built upon functional categories. Because the seven main categories are broken into 26 sub-categories, the resulting score for each clinic may be non-integer. See [18] for more details

As illustrated in Fig. 1, EMR tiered ceiling effect was palpable at the time of our visits in the three settings. It shows what domains of functionalities were not assimilated or could have been assimilated more in each primary care clinic. The three clinics were not reaching maturity level 3 in any domain (see Table 1 for more details about the functionalities associated with maturity levels). Most notably, all clinics were at level 0 in the Evaluation and Monitoring domain that covers health quality indicators, health outcomes, and public health. They were also all at level 1 or below in the domain of Patient Engagement and Communication that covers patient education and self-care/co-management of health.

At Alpha, there was a general feeling that EMR-A could be better assimilated, which would also help realize more clinical benefits such as better patient management: *“The system in itself is very good, it works well, but I’m sure there’s still a lot of untapped potential.” (Nurse 1, Alpha). “[We] should sit down and examine how we could use EMR-A to better serve patients” (Physician 2, Alpha).* Our *de visu* observations allowed us to corroborate users’ comments about the extent to which they used EMR-A to its full potential.

At Delta, the medical director and administrative director felt that assimilation of EMR-B was halted. Some other users were also aware that the clinic was hitting a tiered ceiling in terms of its assimilation of EMR-B, and this was preventing them from attaining the intended benefits:

*“The physicians were all using the EMR’s basic functionalities with no real problems. […] We could become much more productive and improve the quality of the care we give to our patients [by learning EMR-B more].” (Physician 5, Delta)*

Observations of EMR-B in use at the Delta clinic support users’ statements about the degree to which they exploited this EMR’s potential.

At Epsilon, the clinic’s leaders were satisfied with EMR-C since it allowed the clinic to attain its initial objective of reducing paper use. However, several physicians were aware that this objective was very limited in scope, and attainable by simply using the basic functionalities of EMR-C. They acknowledged that they did not know all of the EMR-C’s features: *“I will definitely have to take the time to learn how best to use the EMR.” (Physician 6, Epsilon).* Observations of EMR-C use at the Epsilon clinic corroborated user statements about the extent to which they were using the solution to its full potential. Diagnosis support is one of the domains where Epsilon was at level 0 and could benefit from EMR-C greater assimilation.

### EMR innovation process in the primary care family practices

#### The Alpha family practice

The Alpha primary care clinic had consistently expanded its network over the years and had many facilities to cover its service area. Investing in an EMR became inevitable as a way to support growth and meet the need for real-time information sharing, as well as to follow new computerization trends in the health sector:

*“It was obvious that the records were growing larger and we had no more room. Then the physicians said: ‘We must do something. Now what?’ It was then that we decided to embark on the wave of computerization.” (Administrative Manager, Alpha)*

After taking several part-time university courses on medical informatics, Alpha’s medical director understood all opportunities and benefits offered by IT in general and EMRs in particular. He believed that an EMR could not only deliver administrative benefits and cost savings to the clinic but also be a source of clinical benefits for patients:

*“Sometimes there are projects in health facilities based on questions like: ‘What can we do to improve our conditions as workers?’ But we should be asking: ‘Can we improve patient care?’ If the answer is yes, then that is the direction we should take.” (Medical Director, Alpha)*

Only two EMR solutions were available on the regional market at that time. A physician with a practice near the Alpha clinic had been using EMR-A for several years, and this is where the physician in charge of Alpha saw it for the first time: *“We met the two major vendors at the time. An independent physician in our region used the EMR from one of them, so I visited his practice” (Medical Director, Alpha).* At the same time, the regional health agency obtained financing to computerize the transmission of lab results within its region, and it was the EMR-A supplier who won the tender. The manager of the Alpha clinic then saw the EMR-A solution as their best choice.

The EMR was implemented by the EMR-A supplier working closely with the medical director. He gave information sessions to future users to communicate the upcoming change. Computer savvy, he also conducted several tests of the EMR to demonstrate its reliability. The EMR supplier then provided a short training session that lasted less than a day. Users reported that the lessons learned at this session were quickly forgotten: *“We had a quick training session from the vendor, I would say half a day.” (Physician 1, Alpha).* Both the medical director and the administrative manager were responsible for providing ongoing training and technical support.

Use of the EMR-A system is optional for the physicians but mandatory for the other stakeholders. At the time of data collection the users were relatively satisfied with their EMR since it met the clinic’s administrative objectives as well as some clinical objectives. However, users reported that the clinicians were unable to overcome the ceiling effect for lack of time:

*“[We] should sit down and examine how we could use EMR-A to better serve patients and how EMRs are used elsewhere. We don’t take the time; we’re too caught up in our work.” (Physician 2, Alpha)*

*“At the beginning, we had more medical meetings to talk about our use of the EMR. With time, we have less and less.” (Physician 3, Alpha)*

#### The Delta family practice

The founders of the Delta clinic envisioned a modern, paperless clinic. One of the founders knew the EMR-B’s designer quite well and was convinced that this EMR would allow them to achieve their original vision, which was aligned with recent market trends:

*“It was clear that we would have a paperless clinic. It was a goal to say: ‘We have a paperless clinic.’ One of my colleagues knew an EMR vendor very well. We knew that the computerization of clinics had begun. We went with the flow.” (Physician 1, Delta)*

So no call for tenders was issued, and no requests were made for proposals from other suppliers. All the clinic’s physicians supported the decision to proceed with EMR-B:

*“We felt we had an obligation to choose that vendor because our colleague was close to him. Consequently, we had not really done any research or benchmarking with other EMRs.” (Physician 2, Delta)*

The contract was therefore granted to the EMR-B supplier. The secretaries received a half day of training, which consisted of a demonstration of the software. The physicians were trained by the EMR vendor through a simple demonstration of basic functionalities, but as reported by one of the clinic’s physicians, these lessons were quickly forgotten:

*“I would say that the training from the vendor was not necessarily too quick, but it only covered the basics. It's almost as if I showed you how it works and I begin to click here, click there, click… click… You got the first two clicks and the last two but you missed the other three.” (Physician 3, Delta)*

Despite the poor training, one of the physicians continued to learn about the system and became known by his peers as an advanced EMR user. From his first day at the Delta clinic he used EMR-B daily, exploring its finer points and tailoring it to his practice. He occasionally provided his colleagues with training, sharing his tips on how to be faster and more efficient in their use of the basic EMR functionalities. However, there were fewer and fewer of these training sessions as time went on. Initially, the physicians helped each other use the EMR, but everyone was pressed for time: *“I’d like to ask my colleagues how to use the EMR more effectively, but no-one is available these days…” (Physician 4, Delta).* The technical support for physicians was mainly provided by the administrative manager and, very occasionally, by one of the clinic’s founding physicians.

At the Delta clinic, use of EMR-B was mandatory for all physicians, nurses and administrative staff. Most physicians were relatively satisfied with their EMR use. However, the medical director, administrative director and some users felt that the supplier of EMR-B did not pay sufficient attention to the users’ specific needs at Delta:

*“The supplier had reached the stage where he needed to help us learn [EMR-B] more.” (Physician 5, Delta)*

#### The Epsilon family practice

Due to rapid growth, the Epsilon clinic had begun to run short of space. Physicians’ offices were scattered on many different floors while the medical records were stored in file cabinets close to the secretaries on the first floor. The paper-based records always had to be carried from one floor to another, which made the process cumbersome and inefficient. This situation led the clinical director to decide to invest in an EMR to eliminate paper records and follow market trends:

*“I simply think we are heading towards this digitization. It’s just normal evolution. Because we know it’s coming and it will change medicine. We know we are heading in this direction one way or another. So we had a meeting and we said – ‘Here, we’re going electronic.’” (Physician 1, Epsilon)*

This decision was made easier by the fact that departmental authorities were encouraging computerization initiatives in general in the health network. The medical director and administrative director organized meetings with the leading EMR suppliers for demonstrations of the main products available on the market. However, as reported by one participant, the physicians felt either poorly equipped or unequipped to choose which product to adopt:

*“The biggest problem is that we don’t have any tool to help us select the best EMR. I personally didn’t have the expertise to make such a decision. The vendors simply said, ‘Our EMR product works like this, it does that and that…’” (Physician 2, Epsilon)*

Following these presentations, the clinical director and the administrative coordinator took the initiative to visit some clinics that were using the different EMR solutions to see how they were being used and the main benefits derived.

*“I called the few EMR vendors we had met to know who their customers were. We wanted to see the other clinics and see how they used the software and talk about the perceived benefits. We went by ourselves to visit a few clinics.” (Administrative Manager, Epsilon)*

Following these visits, Epsilon clinic made a decision based on two selection criteria: being able to obtain lab results from hospitals near the clinic and the supplier’s reliability and availability. They finally settled on EMR-C.

The supplier trained the clinic’s secretaries over a period of three non-consecutive days. The content of each training session was determined in collaboration with Epsilon’s administrative director. The physicians were then trained in small groups of two or three according to their schedules. The training consisted of the vendor demonstrating how to use the EMR and, as in the other clinics, these lessons were quickly forgotten:

*“The vendor provided some training, 2-hour sessions. But until you use the system yourself, training doesn’t work. During the training session, the person was showing us how to do things. She had the keyboard and we were watching the screen…” (Physician 3, Epsilon)*

The administrative coordinator, along with a co-worker, was providing ongoing training to users. The physicians were too busy to discuss their EMR use with each other: *“It’s mostly the administrative manager who gives us tricks on how to better use the EMR. Between us physicians, we don’t have time to talk about the system, or how we use it.” (Physician 4, Epsilon)*

EMR use was mandatory for all physicians, nurses and administrative employees. At the start of the assimilation phase, some physicians perceived a negative impact on their relationships with patients during consultations. It was then suggested that each physician would be free to decide whether to use the system before or after each consult rather than during the consult. Medical notes written by hand during the consult were then digitized in the EMR by the clinic’s administrative staff.

In general, the clinic’s leaders were satisfied with how the EMR was being used since it allowed the clinic to attain its initial objectives of gaining space and improving efficiency.

### Constraining conditions on EMR users in the clinics

**Fig. 2:**
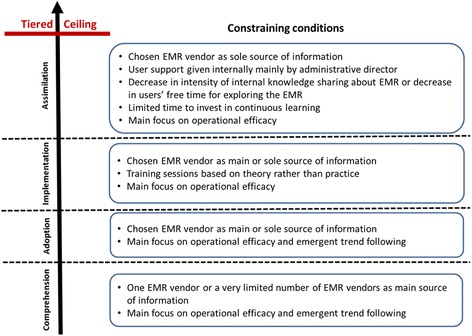
Constraining conditions on EMR assimilation

Figure 2 presents the shared constraining conditions on EMR users, in other words the barriers to EMR assimilation that were present in all three clinics throughout the EMR innovation process.

First, we point that from the beginning to the end of the innovation process, the clinics were all focusing on operational efficiency as the main objective of implementing and using an EMR. Emergent trend following was also an early motivator for the clinics. Clinical objectives that drive the acknowledgement that EMR assimilation must continue once the operational objectives has been attained were absent or secondary in the clinics. This represents an important factor in the appearance of the tiered ceiling effect.

A second interesting observation is that training sessions for EMR users given by the vendors during the implementation phase were based on theory rather than practice in the three clinics. Users were increasingly left to themselves after the training as they saw a decrease in formal and informal knowledge sharing between them about their EMR use or a decrease in individual free time for exploring more advanced functionalities in the EMR. The clinics also had limited time to invest in continuous learning about how to further use their EMRs. After training, the responsibility for user support was taken internally mainly by administrative directors. The clinics felt a slight disengagement from their vendors after the implementation phase, particularly at Delta.

A last but not least interesting observation pertains to information sources about EMRs and their optimal use. Even though at the beginning of the innovation process two of the three clinics received some information about EMRs from the Health Ministry and Agency, and from other clinics, EMR vendors were the main source of information in all the clinics. In the adoption and implementation phases, the chosen EMR vendors became the main or sole source of information. At the end of the innovation process, the chosen EMR vendor had become the sole source of information for each clinic, and was less and less available according to the clinics. According to prior research [21–24, 27], this is an essential factor in the development of a ceiling effect.

In sum, the number of information sources about EMRs had shrunk through the IT innovation process, as well as the number of opportunities for exploring the chosen EMRs and for further learning how to better use them. This general deterioration occurred while the main objective of the clinics towards their EMRs remained operational in nature.

## Discussion

While most research efforts must link results to the literature, it is essential in case study research resting on a limited number of cases to include a comparison of the emergent theory, hypotheses or concepts with the extant literature [38]. This “enfolding” of the literature will further corroborate the generalizability of the results. Our findings generally support the extant literature on ceiling effects [21–24, 27] by showing that limited sources of information and specific users’ parameters are important factors in the occurrence of a tiered ceiling effect. In the following section, we further enfold the literature so to offer plausible explanations for the EMR ceiling effects observed in all three medical practices. We will then present our study limitations and summarize our main contributions.

### Learning within the EMR innovation process

Most physicians we interviewed were not dissatisfied with their EMR per se, but they wanted to assimilate it much further. The vast majority of physicians knew they could do more with their EMR; Fig. 1 shows what domains of functionalities they could have used more. Nevertheless, many considered themselves “good enough” users. They felt that they had less and less time to individually explore the system and continue learning how to make a better use of it. In all the clinics, physicians helped each other learn about the system, but such knowledge sharing mainly occurred at the beginning of the assimilation phase and decreased thereafter. Although some learning had occurred over time, usage evolved slowly and remained rather limited.

Broadly speaking, learning refers to the activity of gaining knowledge carried out by an individual, a group, an organization or a whole community [39]. Knowledge is not a monolithic concept, however; *know-how*, *know-why* and *know-what* are different forms of knowledge [40]. *Know-what* consists of interpretations of what the IT innovation is, i.e. its principles, characteristics and components; *know-why* consists of rationales for IT innovation adoption in organizations; and *know-how* consists of strategies for adopting, implementing and assimilating the IT innovation in organizations. According to Wang and Ramiller [39], knowledge about any IT-based innovation is available in the adopting organization’s environment, i.e. the IT innovation community. This community is usually composed of several entrepreneurs including software vendors and consultants, academics, journalists, industry analysts and adopters [41]. These entrepreneurs participate in the development and evolution of a community discourse about the IT artifact, also referred to as the “organizing vision” [15, 41]. They are the main sources of knowledge available about an IT innovation and they use different means to convey their knowledge, such as conferences or advertisements in magazines [42, 43].

The provincial Health Ministry and regional health agencies served as information sources for the three clinics early in the EMR innovation process, but truly useful information on EMRs was hard to come by from these sources. All three clinics we studied relied heavily on EMR vendors as a source of information throughout the EMR innovation process. This first took the form of presentations by EMR vendors at healthcare industry conferences and/or during vendors’ site visits. Later, it took the form of user training sessions first given during the implementation phase. Thereafter, these sessions were given less and less often and only after major system updates. In all cases, the training provided was rather brief and there were no opportunities for users to try out the system. The format of the training sessions was similar across all the sites and consisted of a demonstration of the EMR performed by a vendor representative. Thus the information gained by users through the EMR innovation process was limited. This shallow knowledge biased physicians toward using only the basic functionalities they could recall*.*

A complex innovation such as an EMR [44] imposes an organizational learning burden that must be addressed in order to lower the knowledge barriers inhibiting its assimilation [45]. According to Argote and Miron-Spektor ([46], p. 1123), “the ability to learn and adapt is critical to the performance and long-term success of organizations.” Given the complexity of EMRs, we posit that organizational learning is a necessary condition for decreasing the likelihood of reaching a ceiling effect in primary care settings. Swanson and Wang [47] showed that for enterprise systems, *know-how* is particularly important in the implementation phase because it favors organizational readiness for change. However, it is not a sufficient condition for IT implementation success; it must be preceded by the appropriate *know-why* during the adoption phase, i.e. the “right” reasons for adoption [47]. This leads us to the phenomenon of “satisficing,” discussed below.

### Satisficing within the EMR innovation process

Before making a choice with regard to a particular innovation, alternatives are usually assessed against several criteria. These are referred to in cognitive theory as “aspirations” (e.g., [48, 49]) and to “motivations” in information systems research [50, 51]. In a study on the adoption of enterprise resource planning (ERP) systems in healthcare organizations, Poba-Nzaou et al. [52] found these motivations to be of three types: business related (gains in operational efficiency), clinical (gains in treatment/care effectiveness), and institutional (gains in overall legitimacy in the field, often demonstrated by trend following).

Using this classification to characterize the primary motivation of each medical practice, it appears that all three clinics wanted to use the EMR to address business-related issues (e.g., more efficient use of space). The rationales developed were heavily based on internal administrative issues at each medical practice. Clinics adopted an institutional perspective to further support their adoption rationale, as key informants mentioned issues like “follow market trends” or “the wave of computerization” when questioned about their impetus to acquire an EMR. At Alpha, however, the clinical perspective (i.e. improving quality of care) was more important than the institutional perspective among the advocates of EMR adoption.

During the adoption phase, each medical practice selected an EMR solution. Our observations show that adoption decisions were aligned with the clinics’ initial motivations. Indeed, clinical *know-why* was virtually nonexistent in the comprehension phase, such that the clinics’ initial motivation could not be refined. During the following implementation phase, the business-oriented perspective was still the main focus in all three clinics. The chosen vendor was therefore fully trusted to install a system that would allow the clinic to reach operational goals such as eliminating paper records. The business-oriented motivation then tainted the assimilation phase of the EMR innovation process, in which no real effort was made to extend the EMR use to reap more benefits. This lack of additional effort can be associated with the principle of “satisficing,” i.e. an option is chosen that meets the specified criteria, even if it is not the optimal one [53].

Such satisficing behavior may be explained by the lack of knowledge (especially *know-why*) that characterized the EMR innovation process in all three clinics. Importantly, the targeted users relied heavily on the EMR vendors for knowledge in each phase and to perform the hands-on tasks during the implementation phase. We believe that this is not specific to the present EMR context. Indeed, software vendors are a predominant source of information for the IT innovations that their clients intend to adopt, especially when the IT is new in a market [39]. However, vendors’ knowledge of how a software solution can be used (*know-how*) may simply translate into the adopter’s knowledge of what it actually does (*know-what*) [39]. In our cases, the vendors may have thought they were transferring practical knowledge (*know-how*) during their training sessions. Yet for the users, these sessions simply covered technical functionalities (*know-what*), most of which were quickly forgotten. Therefore, the EMR users we interviewed were not able to grasp the full potential of their EMR solutions.

It has been found that software vendors tend to present simplified visions of their products to improve their chances of selling them [54], and our three cases confirm this tendency. EMR vendors shape their discourse around the basic goal of any organization implementing an information system: automation of work or “the substitution of machine power for human labor […] for increasing the speed and volume” ([55], p. 6) of work. This discourse emphasizes the administrative benefits of EMRs instead of the clinical ones. This has an impact on the training provided, as it focusses on basic functionalities that minimally disturb existing organizational routines and individual practices. However, to maximize the benefits of EMRs at the community level, more advanced functionalities need to be used, and this requires changes to individual practices [56]. Because these changes have a negative impact on productivity in the short term, most physicians are not interested in investing time and effort in this endeavour.

In sum, we can see that the lack of a formalized and rich discourse about the clinical benefits of adopting an EMR and the tenuous *know-why* linked mainly to administrative efficiency led to satisficing in all three clinics. This lack of knowledge and of further organizational learning persisted throughout the assimilation phase, such that no periodic re-evaluation of EMR use and the related benefits was ever made. This led to the phenomenon of “organizational inertia” that we discuss next.

### Organizational inertia within the EMR innovation process

The ceiling effect observed in all three clinics can also be directly associated with organizational inertia, which is defined as the tendency to commit to current ways of doing things and maintain the status quo in an organization [57]. Inertia creates inflexibilities or rigidities that make it difficult for the organization to efficiently adapt or change [58]. In the IT domain, inertia has been defined as “organizations’ attachment to, and persistence in, using an incumbent system (i.e., the status quo), irrespective of the existence of better [IT-based] alternatives or motivations to change” ([59], p. 4). Alternatively, organizational inertia can be observed when users are not “motivated and able to use the system once it has gone live” ([60], p. 317). In the present study, primary care physicians were able to use their respective EMR after they go-live, but only in basic tasks, for which they relied greatly on their administrative personnel. Satisfied with the outcomes of this basic use, alternative uses involving more advanced EMR functionalities were consciously ignored by the users.

Prior research shows that organizational inertia tends to increase over time [57, 61–63] and that habitual use of an information system enhances this tendency [64, 65]. Habitual use is an automatic behavior that is not re-evaluated by the user unless a major change in the context triggers a strong need for it [66–68]. Habits enable system users to automatically defer to the status quo, ignoring potential alternatives and persisting in habitual use that has already proved satisfactory, efficient and comfortable. In the medical practices we visited, physicians could successfully perform their jobs with minimal use of the EMR.

### Study limitations and contributions

This study has two main limitations that must be acknowledged. First, the data collection was not longitudinal, i.e. we did not follow the IT innovation processes as it unfolded in the three clinics. The interviews were retrospective and recall bias may have tainted our results. Second, we have studied only clinics where a ceiling effect has been attained. It would be useful to verify whether the information sources and users’ characteristics are different in medical clinics where no ceiling effect is observed.

Despite these limitations, we believe that our study offers notable contributions to the current literature. First, whereas Price et al.’s [20] finding on the presence of a ceiling effect is referenced by many, a clear definition of this concept is missing. We propose one which is based on Judson’s [21] definition of the tiered ceiling effect. Second, while Raymond et al. [14, 30] offered an initial hypothetical explanation, we provide a deeper understanding of the elements or conditions that lead to the EMR ceiling effect based on an in-depth multiple case study. Third, we have built on the idea advanced by Yang et al. [3] that studying the entire EMR innovation process is necessary to gain a better understanding of the conditions and contextual issues that stand in the way of beneficial results.

Our explanations for such a ceiling effect are numerous. We first show that clinics are influenced by a public discourse about EMRs coming from a limited number of sources of information that focus on business-related motivations for EMR adoption, such that they set mainly administrative or operational rationales for adoption. These low-level motivations therefore create fertile ground for basic user training, user support given by administrative staff, and low interest in continuous learning and knowledge sharing in subsequent phases of the EMR innovation process. In turn, these conditions constrain the learning of users about more advanced uses of their EMR. Satisficing soon occurs, since the basic knowledge is sufficient to quickly attain the low-level administrative goals related to the clinics’ EMR use. As time passes, satisficing increasingly nourishes habitual use, and this encourages organizational inertia. A lack of incentive for change then fossilizes inertia and inhibits further assimilation and higher-level benefits from EMR use. In sum, these explanations are based on theories and concepts that are not well known in health informatics research, so our contribution goes beyond the frontiers of prior research on EMR innovation and will benefit future studies about the assimilation of other healthcare IT systems.

## Conclusions

The main conclusions of the study are twofold. First, in light of the “satisficing” attitude of many of the physicians interviewed, it appears both important and relevant to reflect on how to favor or encourage continuous learning in primary care medical practices so to overcome ceiling effects in EMR assimilation. While vendor-provided training delivers important information about functionalities and technical details (*know-what*), we suggest that this must be complemented with *know-why* that is directly related to clinical work. This training approach should help physicians refine their adoption rationale and perceive the EMR as a central component of their daily job, not just as another piece of software. Moreover, there is room in the EMR public discourse for clinical benefits. Health authorities and medical associations need to participate more actively in the EMR community discourse to expand the *know-why* component. Because overt learning efforts stop when the desired EMR capability is attained [69], our second conclusion is directly linked to the first. Users who pursue business-related objectives quickly fall into habitual use and become trapped in organizational inertia. This could be, at least partially, alleviated by more training sessions focused on *know-how* (e.g., letting the user control the computer mouse) [70, 71]. This hands-on experience can take the form of a pilot project early in the innovation process, or it can start in the implementation phase. Advanced EMR functionalities can be gradually added to produce the desired clinical benefits. Moreover, since organizational inertia decreases when sufficient information is gathered in the environment for the organization to generate corrective actions [57, 72], users and decision makers need more opportunities to pause and assess the “organizing vision” for EMRs [41]. Maintaining expected organizational routines constrains the ability to imagine alternative uses of information systems and raise the ceiling to the next tier by reflecting on current use and assessing the external environment [44, 73].

## Abbreviations

EMR: Electronic medical record
IS: Information system
IT: Information technology

## Footnotes

1 In this article we use the acronym EMR to refer to the Electronic Medical Record (EMR) system that manages all the patients’ records in one organization. This is coherent with the literature about EMR adoption maturity since using a single record does not bring much benefits.
